# Boron isotope record of peak metamorphic ultrahigh-pressure and retrograde fluid–rock interaction in white mica (Lago di Cignana, Western Alps)

**DOI:** 10.1007/s00410-020-1661-8

**Published:** 2020-02-06

**Authors:** Ralf Halama, Matthias Konrad-Schmolke, Jan C. M. De Hoog

**Affiliations:** 1 School of Geography, Geology and the Environment, Keele University, Keele, ST5 5BG UK; 2 Department of Earth Sciences, University of Gothenburg, Guldhedsgatan 5a, 41320 Gothenburg, Sweden; 3 School of GeoSciences, Grant Institute, James Hutton Road, Edinburgh, EH9 3FE UK

**Keywords:** Boron isotopes, White mica, Subduction, Metamorphism, Fluid–rock interaction, Devolatilization

## Abstract

This study presents boron (B) concentration and isotope data for white mica from (ultra)high-pressure (UHP), subduction-related metamorphic rocks from Lago di Cignana (Western Alps, Italy). These rocks are of specific geological interest, because they comprise the most deeply subducted rocks of oceanic origin worldwide. Boron geochemistry can track fluid–rock interaction during their metamorphic evolution and provide important insights into mass transfer processes in subduction zones. The highest B contents (up to 345 μg/g B) occur in peak metamorphic phengite from a garnet–phengite quartzite. The B isotopic composition is variable (δ^11^B = − 10.3 to − 3.6%) and correlates positively with B concentrations. Based on similar textures and major element mica composition, neither textural differences, prograde growth zoning, diffusion nor a retrograde overprint can explain this correlation. Modelling shows that B devolatilization during metamorphism can explain the general trend, but fails to account for the wide compositional and isotopic variability in a single, well-equilibrated sample. We, therefore, argue that this trend represents fluid–rock interaction during peak metamorphic conditions. This interpretation is supported by fluid–rock interaction modelling of boron leaching and boron addition that can successfully reproduce the observed spread in δ^11^B and [B]. Taking into account the local availability of serpentinites as potential source rocks of the fluids, the temperatures reached during peak metamorphism that allow for serpentine dehydration, and the high positive δ^11^B values (δ^11^B = 20 ± 5) modelled for the fluids, an influx of serpentinite-derived fluid appears likely. Paragonite in lawsonite pseudomorphs in an eclogite and phengite from a retrogressed metabasite have B contents between 12 and 68 μg/g and δ^11^B values that cluster around 0% (δ^11^B = − 5.0 to + 3.5). White mica in both samples is related to distinct stages of retrograde metamorphism during exhumation of the rocks. The variable B geochemistry can be successfully modelled as fluid–rock interaction with low-to-moderate (< 3) fluid/rock ratios, where mica equilibrates with a fluid into which B preferentially partitions, causing leaching of B from the rock. The metamorphic rocks from Lago di Cignana show variable retention of B in white mica during subduction-related metamorphism and exhumation. The variability in the B geochemical signature in white mica is significant and enhances our understanding of metamorphic processes and their role in element transfer in subduction zones.

**Electronic supplementary material:**

The online version of this article (10.1007/s00410-020-1661-8) contains supplementary material, which is available to authorized users.

## Introduction

Boron (B) and the B stable isotope system are useful tracers of fluid-mediated mass transfer in subduction zones. Boron elemental and isotopic data can be used to track water cycling and the origin of fluid sources in subduction zone magmatism (Marschall et al. 2007; Konrad-Schmolke and Halama 2014; De Hoog and Savov 2018; Palmer 2017) as well as fluid–rock interaction processes and metasomatism in the metamorphic evolution of subduction-related metamorphic rocks (Bebout and Nakamura 2003; Marschall et al. 2006b, 2009; Halama et al. 2014). Across-arc profiles in arc lavas show a systematic and coupled decrease in B concentrations and B isotopic compositions (Ishikawa and Nakamura 1994; Ishikawa et al. 2001). These trends have been attributed to a decreasing addition of slab-derived fluids with increasing slab depth (Bebout et al. 1999; Peacock and Hervig 1999; Rosner et al. 2003) based on the preferential partitioning of B into the fluid (Brenan et al. 1998) and the large isotopic fractionation of the two stable B isotopes (^11^B and ^10^B) with a relative preference of the heavy isotope ^11^B for the fluid (Wunder et al. 2005; Sanchez-Valle et al. 2005). Consequently, slab dehydration is expected to lead to successively decreasing δ^11^B values in the dehydrating rocks of the subducting slab (Moran et al. 1992; Marschall et al. 2007; Konrad-Schmolke and Halama 2014). Whole-rock analyses of high-pressure metamorphic rocks have also shown that B correlates positively with H_2_O contents and traces progressive dehydration (Marschall et al. 2009; Scambelluri et al. 2004), which can be used to model across-arc variations in volcanic rocks that show systematic trends in B geochemistry (Konrad-Schmolke et al. 2016).

Several studies have focused on tourmaline as the major B-hosting phase to track the B isotopic evolution of fluids in subduction zone settings (Bebout and Nakamura 2003; Marschall et al. 2006b; Ota et al. 2008). However, as tourmaline is absent in many lithologies, white mica (phengite, muscovite and/or paragonite) can act as a major mineral host for B, which substitutes for tetrahedrally coordinated aluminium (Wunder et al. 2005). Since white mica is stable over a wide range of temperatures and pressures in subduction-related metamorphic rocks and common in both meta-igneous and metasedimentary rocks, it is a suitable alternative to investigate B systematics (e.g. Peacock and Hervig 1999; Pabst et al. 2012; Trumbull and Slack 2018). Subsequent work showed that the complex interplay of dehydration and rehydration during exhumation, deformation and metasomatic events could be traced in zoned white mica from subduction-related rocks utilizing B as an indicative element (Konrad-Schmolke et al. 2011; Angiboust et al. 2014; Halama et al. 2014; Sievers et al. 2017).

In this study, we investigate the B concentrations and B isotope composition of white mica in (ultra)high-pressure (UHP) metamorphic rocks from Lago di Cignana (Western Alps, Italy). We investigate three different lithologies—a garnet–phengite quartzite, an eclogite and a retrogressed metabasite—that have experienced a similar P/T evolution in a subduction zone setting. Fluid–rock interaction models successfully match the data for both peak metamorphic and retrogressed samples, demonstrating that boron measurements in white mica are a sensitive probe into fluid–rock interaction processes and emphasizes the role of intergranular fluids for element mobility and transfer even at ultrahigh-pressure metamorphic conditions.

## Geological setting

**Fig. 1:**
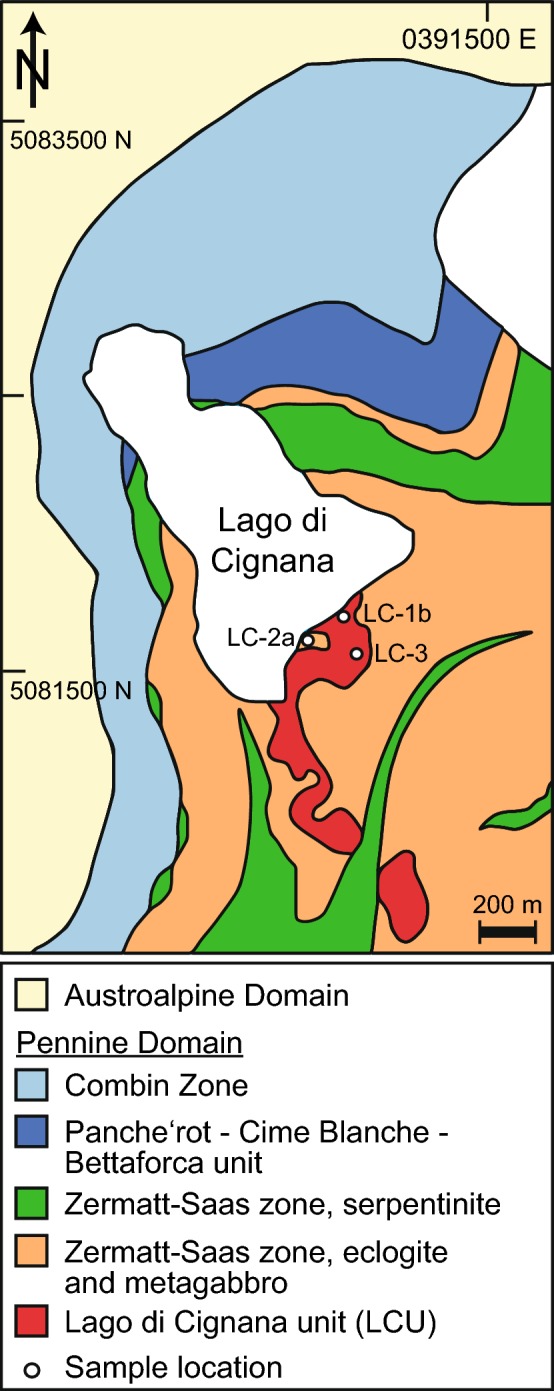
Simplified geological map of the Lago di Cignana area (after Groppo et al. 2009) with sample locations

A belt of subduction-related (U)HP metamorphic rocks in the Western Alps stretches from the Mediterranean Sea to Switzerland (Fig. 1). These rocks are part of the Penninic Domain in the Alps, which represent remnants of the Tethys Ocean (or Piemonte-Liguria Ocean) between the European continent to the NW and the Apulia plate to the SE (Dal Piaz 1974). The Penninic Domain comprises igneous oceanic crust of the Tethys Ocean (Piemonte-Liguria Ocean) and the sedimentary cover that was deposited along its European continental margin (Beltrando et al. 2010). The subduction-related (U)HP rocks underwent Alpine metamorphism and constitute the Piemonte Zone, which can be subdivided into an eclogite-facies Zermatt-Saas Zone and a greenschist- to blueschist-facies Combin Zone (Fig. 1). In the Zermatt-Saas Zone, bodies of Fe-Ti gabbros converted to eclogites and Mg–Al gabbros occur with serpentinized mantle peridotites. The serpentinites are locally overlain by metasediments (calcschists and impure quartzites) and/or pillow lavas representing meta-ophiolitic lithologies (Beltrando et al. 2010). Peak metamorphic pressures were reached at 48–44 Ma before juxtaposition of the Zermatt-Saas with the Combin Zone at around 38 Ma (Rubatto et al. 1998; Reddy et al. 1999; Lapen et al. 2003; Beltrando et al. 2009).

At the Lago di Cignana in the Valtournenche, NW Italy (Fig. 1), tectonic slices of meta-ophiolites of the Zermatt-Saas Zone enclose a coesite- and diamond-bearing UHP metamorphic unit, the Lago di Cignana Unit (LCU) (Pleuger et al. 2007; Groppo et al. 2009; Frezzotti et al. 2011). The lithologies of the LCU represent former oceanic crust and comprise metabasites and metasedimentary rocks (Compagnoni and Rolfo 2003). Metabasites of the LCU are geochemically similar to those of the Zermatt-Saas Zone, showing a typical mid-ocean ridge basalt (MORB) signature with an oceanic tholeiitic parentage. The metasedimentary rocks comprise impure marbles and quartzites, calcschists and Mn-rich garnetites with Fe–Mn nodules (Bearth 1967; Dal Piaz et al. 1979). Garnets from these metasedimentary rocks contain microdiamond inclusions (Frezzotti et al. 2011, 2014).

The prograde metamorphic evolution of the LCU was reconstructed for eclogites and metasedimentary rocks based on mineral growth zoning and mineral inclusions in garnet (Reinecke 1991; van der Klauw et al. 1997). Prograde garnet growth in eclogites lasted for 12 Myears reflecting the duration of slab subduction to UHP conditions at depths > 90 km (Lapen et al. 2003). Peak metamorphic conditions reached 615 ± 15 °C and 2.8 ± 1.0 GPa (Reinecke 1991, 1998). Some greenschist-facies retrogression occurs in the eclogites of the LCU (van der Klauw et al. 1997). The peak metamorphic conditions of the LCU overlap with those of eclogite-facies rocks from the Zermatt-Saas Zone (550–600 °C and 2.5–3.0 GPa). Groppo et al. (2009) have shown that pressure estimates from the meta-ophiolites of the LCU and the Zermatt-Saas Zone differ by less than 0.3 GPa, and both units have identical compositions and zoning patterns of major minerals. Thus, the presence/absence of coesite is the only discriminating factor (Groppo et al. 2009). U–Pb dating of metamorphic zircon and ^40^Ar/^39^Ar dating of phengite from the LCU gave ages around 44 Ma (Rubatto et al. 1998; Gouzu et al. 2006), similar to those obtained for the Zermatt-Saas Zone and hence supporting a similar tectonometamorphic evolution.

## Samples

**Fig. 2:**
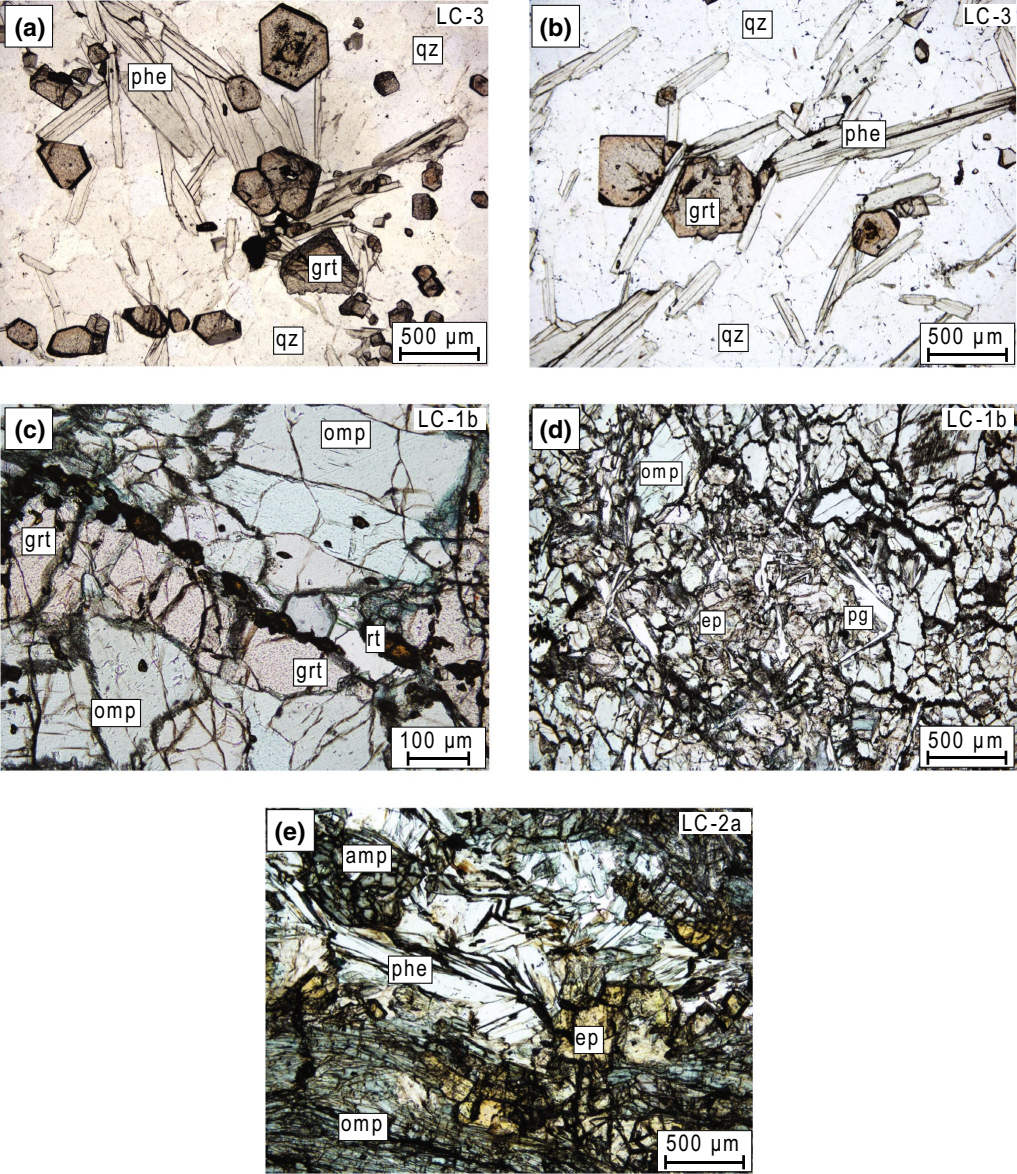
Representative thin-section images of the (U)HP samples from Lago di Cignana. **a**, **b** Garnet–phengite quartzite. **c**, **d** Eclogite. **e** Retrogressed metabasite. *Grt* garnet, *phe* phengite, *qz* quartz, *omp* omphacite, *rt* rutile, *ep* epidote, *pg* paragonite, *amp* amphibole

Three samples from the HP/UHP units around Lago di Cignana were investigated (Fig. 2): (1) a fine-to-medium-grained garnet–phengite quartzite (sample LC-3: 45.8770° N, 7.5933° E) of the LCU contains quartz, garnet and phengite as major mineral phases. The mostly randomly oriented, interlocking or discrete elongated flakes of phengite define a decussate texture. Quartz grains are anhedral with irregular outlines. Euhedral garnet is typically 100–500 μm in diameter and zoned. Typical garnet zoning shows faintly pink-coloured cores, relatively enriched in Mn and Mg, and reddish-brownish rims that are richer in Fe and Ca. Minor amphibole and biotite are present, and rutile and opaques occur as accessory phases. (2) A weakly foliated eclogite (sample LC-1b: 45.8786° N, 7.5927° E) from the LCU, which is fine-to-medium-grained and comprises garnet, omphacite, blue amphibole, epidote, paragonite and quartz as major mineral phases. Euhedral garnet is porphyroblastic and reaches up to 3 mm in diameter. Retrograde chlorite along fractures in garnet is occasionally present but rare, and incipient replacement of blue by green amphibole at the crystal rims is also observed. Accessory phases are rutile and apatite. Paragonite is sometimes associated with rectangular to rhombic outlines of a precursor phase (Fig. 2), which has been interpreted as representing pseudomorphs after lawsonite forming in an early post-peak metamorphic phase during decompression (Groppo et al., 2009). (3) A strongly foliated, fine-grained metabasite (sample LC-2a: 45.8776° N, 7.5918° E) shows a pervasive retrogression into a greenschist-facies mineral assemblage. This rock belongs to the Zermatt-Saas Zone and is attributed to the upper unit of Groppo et al. (2009). Omphacite, phengite, epidote/clinozoisite, albite and chlorite are the major mineral phases. Amphibole is a minor phase, and titanite and rutile occur as accessory phases.

## Analytical methods

Wavelength-dispersive quantitative mineral chemical analyses were performed at the University of Kiel (Germany) using a JEOL JXA 8900R electron microprobe. Elements were measured 15 s on peak and 7 s on background with a beam diameter of 5 μm, a beam current of 15 nA and an acceleration voltage of 15 kV. Natural standards were used for calibration and a CITZAF matrix correction was applied. Secondary standards used for quality control were garnet (Roberts Victor 2; USNM 87375), plagioclase (Lake County labradorite; USNM 115900) and olivine (Springwater forsterite 83; USNM 2566) from the Smithsonian Institution, Washington DC (Jarosewich et al. 1980). Element distribution maps were acquired with a Hitachi TM3000 scanning electron microscope at Keele University (see electronic supplementary material).

Boron concentration and isotope analyses were performed at the Edinburgh Ion Microprobe Facility (EIMF) in Edinburgh using a Cameca IMS-1270 ion microprobe. Gold-coated thin sections were sputtered by an 8 nA ^16^O_2_^−^ primary beam with a net impact energy of 22.5 keV in Kohler illumination mode. Sputter pit diameter was ca. 20 × 30 µm. Mass resolution (ΔM/M) was ~ 2400 to avoid interferences from ^9^BeH^+^ and ^10^BH^+^. At the start of each analysis, the analytical area was pre-sputtered for 60 s using the same beam conditions as during the measurement itself. Subsequently, using an automated routine, the secondary beam was centred relative to the field aperture and the mass centre of the ^11^B peak was determined. Each analysis consisted of 50 cycles during which ^10^B and ^11^B signals were counted sequentially by an electron multiplier detector with counting times of 8 s and 2 s per cycle, respectively. ^11^B signals were divided by time-averaged ^10^B signals of the cycles before and after, resulting in 49 ^11^B/^10^B ratios per analysis. The ^11^B count rate was ca. 180 cps na^−1^ μg/g^−1^. One second uncertainties are reported in per mil as relative standard error, i.e., the relative standard deviation of all analytical cycles divided by the square root of the total number of cycles. Raw ^11^B/^10^B ratios were converted to true ^11^B/^10^B ratios based on reference standards measured at regular intervals throughout the analytical session (see details below) and converted to delta notation (δ^11^B, per mil deviation from reference standard NIST SRM951) using a value of 4.04362 (Catanzaro et al. 1970). The calibration slope (instrumental mass fractionation) ranged from 0.937 to 0.952 during the session. Boron concentrations were estimated based on ^11^B count rates of samples and silicate glass reference materials.

The following reference standards were used (Table 1): glasses GOR128-G komatiite and StHs6/80-G dacite (Rosner and Meixner 2004), phengite 80-3 (Pabst et al. 2012), and micas MVE02-8-5 and JJE01-X-3 (Martin et al. 2015). A small offset in δ^11^B of − 1.2% for phengite 80-3 was observed compared to the glasses, in agreement with offset of micas relative to anhydrous glasses reported by Pabst et al. (2012) and De Hoog et al. (2017). Matrix-induced fractionation is often present in SIMS and matrix matching is, therefore, essential, as the physics behind the fractionation are poorly understood (Eiler et al. 1997; Rosner et al. 2008). Accordingly, mica data reported in this study were also corrected by − 1.2% relative to the glass standards. Mica MVE02-8-5 has an offset of − 4.0%, which is larger than Phe 80-3, but the published values vary by nearly 3%, making the material unsuitable for calibration. Mica JJE01-X-3 had a large offset of nearly + 7% compared to the other two micas, which we cannot explain.

**Table 1:** Boron isotope data of measured reference standards

Standard	δ^11^B (glass) (%)	δ^11^B (mica) (%)	1 s (%)	*n*	δ^11^B ± 1 s (ref) (%)	*B* (ppm)	1 s	*B* (ref) (ppm)
StHs6/80-G	− 4.6		± 0.3	16	− *4.39* ± *0.13*	12.5	± 0.4	*11.6*^a^
GOR128-G	+ 13.8		± 0.4	16	+ *13.55* ± *0.11*^a^	20	± 3	*22.7*^a^
GSD1-G	+ 10.2		± 0.1	25	+ *10.2* ± *0.25*^b^	67	± 4	*50* ± *20*^b^
Mica MVE02-8–5	− 6.6	− 5.4	± 0.2	14	− *2.6* ± *1.7*^c^	73	± 8	*60–140*^c^
Phengite 80–3	− 14.7	− 13.5	± 0.9	7	− *13.50* ± *0.35*^d^	38	± 8	*27.1d*^c^
Mica JJE01-X-3	− 0.7	+ 0.5	± 0.3	8	− *6.3* ± *0.6*c^d^	291	± 48	*30–300*^c^

Numbers in italics are reference (ref) values

1 s uncertainty of δ^11^B is standard error of the mean of *n* repeat measurements

^a^Rosner and Meixner (2004)

^b^Jochum et al. (2011)

^c^Martin et al. (2015, 2016)

^d^Pabst et al. (2012)

Each set of analyses of the samples was bracketed by 4–6 analyses of basaltic glass GSD1-G and mica MVE02-8-5 to monitor drift and matrix-dependent fractionation. The remaining glass and mineral standards were measured several times during the analytical session. The precision of the analyses was evaluated as follows: the internal uncertainty was based on the 50 repeat cycles of each analyses, and ranges from 0.3 to 0.9% depending on the B concentration of the sample. The drift correction added 0.23% based on the uncertainty of measurements of bracketing standards MVE02-8-5 and GSD1-G, and it presented as external precision in Tables 2, 3 and 4.

**Table 2:** Major element and boron chemistry of white mica from the garnet phengite quartzite (sample LC-3)

SIMS spot number	18	19	20	21	22	23	24	25
Wt%								
SiO_2_	51.16	51.33	50.72	51.18	51.13	51.18	51.06	51.46
TiO_2_	0.22	0.17	0.15	0.15	0.27	0.15	0.12	0.29
Al_2_O_3_	25.01	24.06	25.12	23.70	24.44	23.75	24.79	24.08
Cr_2_O_3_	0.03	0.02	0.03	b.d.l	0.03	b.d.l	b.d.l	0.07
FeO	4.01	4.04	4.06	4.11	4.15	4.04	3.89	4.10
MnO	0.07	0.08	0.06	0.04	0.07	0.03	0.07	0.04
MgO	3.42	3.90	3.34	3.70	3.55	3.62	3.48	3.66
CaO	0.02	b.d.l	b.d.l	b.d.l	b.d.l	0.08	b.d.l	b.d.l
Na_2_O	0.45	0.22	0.35	0.19	0.33	0.23	0.36	0.21
K_2_O	10.98	11.19	11.01	11.22	11.20	11.04	10.90	11.31
Total	95.37	95.01	94.83	94.29	95.17	94.11	94.68	95.22
Cations per formula unit								
Si	6.910	6.967	6.893	7.001	6.935	7.006	6.937	6.974
Al	3.981	3.849	4.023	3.821	3.907	3.832	3.969	3.846
Ti	0.022	0.018	0.015	0.016	0.027	0.015	0.013	0.029
Cr	0.003	0.002	0.003	–	0.003	–	–	0.008
Mg	0.689	0.789	0.677	0.755	0.718	0.739	0.705	0.739
Fe	0.453	0.459	0.461	0.470	0.471	0.463	0.442	0.465
Mn	0.008	0.009	0.007	0.004	0.009	0.003	0.008	0.004
Ca	0.003	–	–	–	–	0.012	–	–
Na	0.118	0.059	0.091	0.051	0.087	0.060	0.095	0.056
K	1.892	1.938	1.909	1.958	1.938	1.928	1.889	1.955
Total	14.080	14.088	14.079	14.077	14.095	14.057	14.058	14.076
Mg#	0.603	0.632	0.595	0.616	0.604	0.615	0.615	0.614
Na/(Na+K)	0.059	0.030	0.046	0.025	0.043	0.030	0.048	0.028
Boron data								
B (μg/g)	113	240	194	179	151	345	109	263
δ^11^B (%)	− 10.2	− 6.4	− 6.4	− 6.5	− 9.7	− 3.6	− 10.3	− 5.2
External precision ± 1 s (%)	0.6	0.6	0.9	0.5	0.6	0.6	0.5	0.6
Accuracy ± 1 s (%)	1.8	1.8	2.1	1.8	1.8	1.8	1.7	1.8

*b.d.l.* below detection limit

**Table 3:** Major element and boron chemistry of white mica from the eclogite (sample LC-1b)

SIMS spot number	162	163	164	165	166	167	168
Wt%							
SiO_2_	47.12	47.60	47.53	47.54	47.40	47.65	47.76
TiO_2_	0.06	0.09	0.06	b.d.l	0.04	0.09	0.06
Al_2_O_3_	38.67	38.09	38.56	38.96	38.26	39.04	38.13
Cr_2_O_3_	0.01	0.03	0.03	b.d.l	b.d.l	0.04	0.01
FeO	0.23	0.33	0.34	0.22	0.40	0.18	0.32
MnO	0.01	b.d.l	0.02	b.d.l	0.03	0.01	b.d.l
MgO	0.16	0.32	0.27	0.16	0.26	0.20	0.54
CaO	0.21	0.18	0.15	0.19	0.21	0.16	0.17
Na_2_O	7.79	7.74	7.91	8.11	7.93	8.12	7.93
K_2_O	0.43	0.73	0.55	0.40	0.55	0.34	0.78
Total	94.69	95.10	95.42	95.58	95.08	95.82	95.70
Cations per formula unit							
Si	6.055	6.102	6.071	6.056	6.080	6.052	6.092
Al	5.857	5.755	5.805	5.850	5.784	5.844	5.732
Ti	0.006	0.008	0.006	–	0.004	0.009	0.006
Cr	0.001	0.003	0.003	–	–	0.004	0.001
Mg	0.031	0.061	0.052	0.030	0.050	0.037	0.102
Fe	0.024	0.036	0.036	0.023	0.042	0.019	0.034
Mn	0.001	–	0.003	–	0.003	0.001	–
Ca	0.029	0.024	0.020	0.026	0.029	0.022	0.023
Na	1.941	1.924	1.959	2.003	1.972	2.000	1.961
K	0.071	0.120	0.089	0.065	0.090	0.055	0.127
Total	14.016	14.033	14.043	14.053	14.055	14.042	14.079
Mg#	0.558	0.629	0.592	0.562	0.539	0.660	0.748
Na/(Na+K)	0.965	0.941	0.956	0.969	0.957	0.973	0.939
Boron data							
B (μg/g)	12.3	19.7	22.5	68.1	13.9	14.6	23.8
δ^11^B (%)	− 4.2	− 2.5	− 1.7	2.8	0.0	− 5.0	− 3.9
External precision ± 1 s (%)	1.1	0.9	0.9	0.6	1.1	1.0	1.0
Accuracy ± 1 s (%)	2.3	2.1	2.1	1.8	2.3	2.2	2.2

*b.d.l.* below detection limit

**Table 4:** Major element and boron chemistry of white mica from the retrogressed metabasite (sample LC-2a)

SIMS spot number	235	236	237	238	239	240	241	242	243
Wt%									
SiO_2_	50.58	50.27	50.37	49.78	50.35	51.55	50.63	52.68	50.85
TiO_2_	0.13	0.14	0.32	0.15	0.25	0.22	0.08	b.d.l	0.18
Al_2_O_3_	27.45	28.25	28.20	28.45	27.60	27.00	27.78	24.33	27.41
Cr_2_O_3_	0.54	0.53	0.59	0.46	0.57	0.42	0.50	0.92	0.84
FeO	1.63	1.75	1.59	1.65	1.79	1.26	1.68	0.93	1.34
MnO	0.05	0.05	0.01	b.d.l	b.d.l	0.02	0.02	0.03	0.03
MgO	3.39	3.09	3.35	3.14	3.29	3.65	3.37	4.80	3.53
CaO	b.d.l	b.d.l	b.d.l	b.d.l	0.02	b.d.l	b.d.l	b.d.l	b.d.l
Na_2_O	0.82	0.88	0.89	0.92	0.79	0.70	0.83	0.28	0.83
K_2_O	10.52	10.41	10.32	10.50	10.33	10.71	10.49	11.34	10.53
Total	95.11	95.37	95.64	95.04	94.99	95.54	95.37	95.31	95.53
Cations per formula unit									
Si	6.765	6.706	6.694	6.667	6.741	6.845	6.750	7.027	6.766
Al	4.327	4.441	4.417	4.491	4.355	4.225	4.365	3.825	4.298
Ti	0.013	0.014	0.032	0.015	0.025	0.022	0.008	–	0.018
Cr	0.057	0.056	0.062	0.049	0.061	0.045	0.052	0.097	0.088
Mg	0.676	0.614	0.664	0.627	0.657	0.722	0.670	0.955	0.700
Fe	0.182	0.195	0.177	0.185	0.200	0.140	0.187	0.104	0.149
Mn	0.006	0.006	0.001	–	–	0.002	0.002	0.003	0.003
Ca	–	–	–	–	0.002	–	–	–	–
Na	0.212	0.228	0.230	0.238	0.205	0.180	0.213	0.072	0.213
K	1.795	1.772	1.750	1.794	1.764	1.814	1.784	1.930	1.787
Total	14.034	14.032	14.025	14.065	14.011	13.996	14.032	14.013	14.023
Mg#	0.788	0.759	0.790	0.772	0.766	0.838	0.781	0.902	0.824
Na/(Na+K)	0.106	0.114	0.116	0.117	0.104	0.090	0.107	0.036	0.107
Boron data									
*B* (μg/g)	44.4	67.7	33.5	29.0	39.1	52.5	37.1	57.1	32.2
δ^11^B (%)	− 4.1	− 1.6	− 0.6	− 2.3	− 1.3	3.1	− 1.1	3.5	0.9
External precision ± 1 s (%)	0.9	0.7	0.8	0.8	0.7	0.7	0.8	0.7	0.9
Accuracy ± 1 s (%)	2.1	1.9	2.1	2.0	1.9	1.9	2.0	1.9	2.1

*b.d.l.* below detection limit

The accuracy of the analyses is dependent on the uncertainty of the standards used to determine the matrix-dependent mass fractionation. This uncertainty has two components: the measurement uncertainty and the uncertainty of the literature value. The latter is small for phengite 80-3 (0.35%; Pabst et al. 2012), but the MVE02-8-5 mica standards suffer from a large uncertainty (1.7%; after Martin et al. 2016), even though our material is homogeneous within counting statistics, suggesting heterogeneity between different batches of the material. In contrast, our phengite 80-3 standard is clearly inhomogeneous, resulting in a relative large uncertainty of 0.9% for seven repeats (Table 1). Nevertheless, the cumulative uncertainty for Phe 80-3 (1.2%) is much smaller than that of MVE02-8-5 and, therefore, we only used Phe 80-3 for matrix-dependent mass fractionation correction, in line with the recent literature (Pabst et al. 2012; Angiboust et al 2014). The uncertainty in the accuracy is presented separately in Tables 2, 3 and 4, as it is only applicable when comparing our results to B isotope data from the literature.

## Results

### Mineral chemistry of white mica

**Fig. 3:**
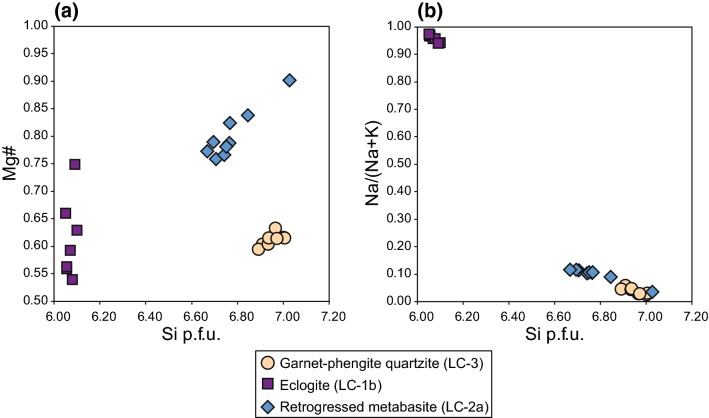
Major element mineral chemistry of white mica from the Lago di Cignana (U)HP rocks. **a** Mg# vs Si p.f.u. and **b** Na/(Na+K) vs Si p.f.u. White mica is phengite in samples LC-3 and LC-2a and paragonite in sample LC-1b

The investigated white micas show distinct compositional differences based on Mg# (Mg# = Mg/[Mg+Fe^2+^]), Na/(Na+K) and Si contents (Fig. 3; Tables 2, 3 and 4; cations calculated on the basis of 22 oxygens). Element distribution maps are provided in the electronic supplementary material.

In the garnet–phengite quartzite (LC-3), the compositional variability of phengite is small with Mg# covering a range from 0.59 to 0.63. Na/(Na+K) ranges from 0.03 to 0.06 and Si per formula unit (p.f.u.) varies between 6.89 and 7.01. Mg# and Si p.f.u. are positively correlated in phengite. Individual phengite grains are homogeneous and do not show any visible zonation in major elements (supplementary Figs. 1, 2). Adjacent but distinct phengite grains also lack any significant differences in major element composition.

The eclogite (sample LC-1b) contains only paragonite but no phengite, displaying small variations in Na/(Na+K) (0.94–0.97) and Si p.f.u. (6.05–6.10). Mg# in paragonite is variable, covering a range from 0.54 to 0.75. However, concentrations in both MgO (0.16–0.54 wt%) and FeO (0.18–0.40 wt%) are low, so that small variations in the concentrations have relatively large effects on Mg#. Overall, variations in major element contents are small and element distribution maps show compositionally homogeneous grains (supplementary Fig. 3).

Phengite in the retrogressed metabasite (sample LC-2) is chemically more variable than white mica in the other two samples for both Si p.f.u. (6.67–7.03) and Na/(Na+K) (0.04–0.12). Mg# varies from 0.76 to 0.90 and correlates positively with Si p.f.u., similar to phengite in the garnet–phengite quartzite. Element distribution maps reveal a patchy zonation in Si, Al and Mg (supplementary Fig. 4). Areas of higher Mg# coincide with elevated Si contents, but the spatial distribution of these areas relative to the grain boundaries is not well defined.

### Boron elemental and isotopic compositions of white mica

**Fig. 4:**
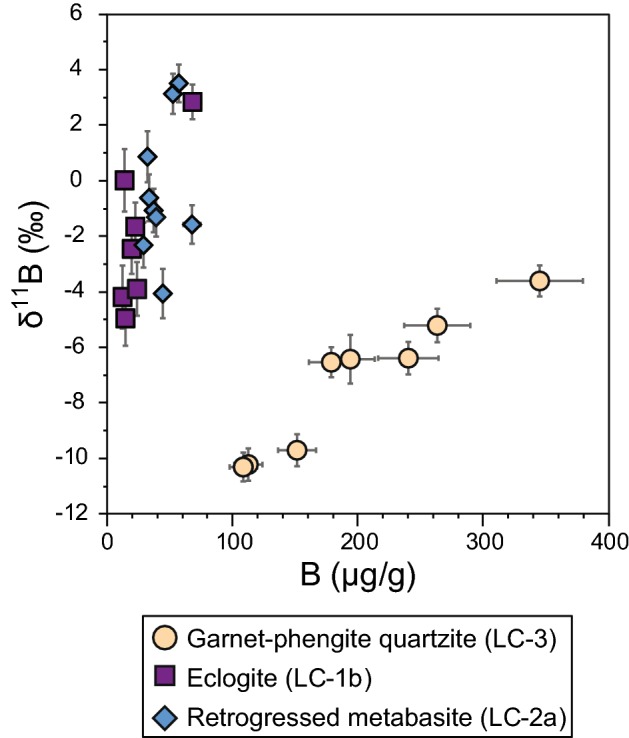
Boron geochemistry systematics of white mica from the Lago di Cignana (U)HP rocks. Error bars are ± 10% for [B] and the external precision of each spot for δ^11^B (see Tables 2, 3, 4)

The diagram δ^11^B versus B content (Fig. 4) illustrates the key features of boron geochemistry in white mica of the investigated samples (Tables 2, 3, 4). Boron concentrations [B] in phengite from the garnet–phengite quartzite (sample LC-3, beige circles) are relatively high compared to the mafic rocks and range from 109 to 345 μg/g. δ^11^B varies from − 10.3 to − 3.6%, showing a very distinct positive correlation between δ^11^B and [B]. Paragonite in the eclogite (sample LC-1b, purple squares) exhibits a range in [B] from 12 to 68 μg/g, with six out of seven analyses yielding < 25 μg/g B. δ^11^B varies from − 5.0 to + 2.8%, but a clear correlation between [B] and δ^11^B is lacking. Phengite in the retrogressed metabasite (sample LC-2a; blue diamonds) has B contents in the range 29–57 μg/g and δ^11^B from − 4.1 to + 3.5. These values show considerable overlap with the B geochemistry of the eclogite (sample LC-1b).

## Discussion

### Effects of protolith geochemistry

Differences in the B contents between white mica from the metabasic rocks (12–68 μg/g B) and the metasedimentary garnet–phengite quartzite (113–345 μg/g B) may reflect the relative B enrichment of sedimentary compared to mafic igneous protoliths. For the metabasic rocks, mafic rocks of the oceanic crust represent protolith lithologies. Boron contents in MORBs range from 0.4 to 2.5 μg/g (Marschall et al. 2017) and whole-rock data for bulk gabbros, dolerites and basalts from the Oman ophiolite vary from < 1 to 29 μg/g B (Yamaoka et al. 2012). In comparison, bulk B concentrations in marine sediments are typically higher, ranging from 30 to 120 μg/g in shales (Romer et al. 2014) and from 52 to 100 μg/g in turbidites (Leeman et al. 2004). Siliceous ooze and chert, lithologies that can be taken as potential protoliths to the quartzite, have 35–97 μg/g B (Ishikawa and Nakamura 1993; Kolodny and Chaussidon 2004). Hence, cherts are roughly 10–100 times enriched in B compared to typical mafic rocks of the (altered) oceanic crust. Even though the Cignana metabasites are low in K_2_O (0.11–0.29 wt%; Groppo et al. 2009), HP quartzites (1.55 wt% K_2_O; Selverstone and Sharp 2013) are approximately only 10 × enriched compared to the metabasites. Therefore, the higher B_phengite_ contents in the garnet–phengite quartzite are consistent with higher [B] in the protolith. Published B concentration data for white mica in metabasic rocks include phengite in blueschist fragments from the Mariana forearc with 29–50 μg/g B (Pabst et al. 2012) as well as phengite and paragonite in metabasic HP rocks from Syros (Greece) with 43–136 μg/g B (Marschall et al. 2006a) and from Guatemala with 0.7–165 μg/g (Martin et al. 2016), overlapping with the values measured in the Lago di Cignana metabasites. In contrast, boron concentrations in white mica from metasedimentary rocks are highly variable but can reach up to 5500 μg/g in continental crustal HP rocks (Sievers et al. 2017).

For potassic white mica, such as phengite, the K_2_O content of the bulk rock also influences the B concentration in phengite, because phengite is the major host of both K_2_O and B in these type of rocks (Bebout and Fogel 1992; Bebout et al. 2007). Therefore, the ratio B/K_2_O in phengite is directly proportional to the B/K_2_O ratio in the bulk rock. Since K_2_O in phengite is essentially fixed to ca. 9–11 wt% due to crystal chemical controls, protoliths with low K_2_O content would crystallize phengite with high B contents as [B]_phengite_ ∝ [B]_rock_/[K_2_O]_rock_.

### White mica boron geochemistry

Processes that can cause variability in the boron geochemistry of white mica from HP and UHP metamorphic rocks include (1) inhomogeneities of the protolith, (2) retrograde overprint, (3) prograde growth zoning, (4) diffusion, (5) prograde devolatilization with loss of volatile elements, (6) peak metamorphic fluid–rock interaction (Barrientos and Selverstone 1993; Halama and Konrad-Schmolke 2015). In the following, we will first discuss these processes with regard to the garnet–phengite quartzite. For both the eclogite and the retrogressed metabasite, petrographic observations show that white mica formed during retrograde metamorphism, and their boron geochemistry will be evaluated accordingly.

#### Boron elemental and isotopic systematics in the garnet–phengite quartzite

##### Inhomogeneity of the protolith

**Fig. 5:**
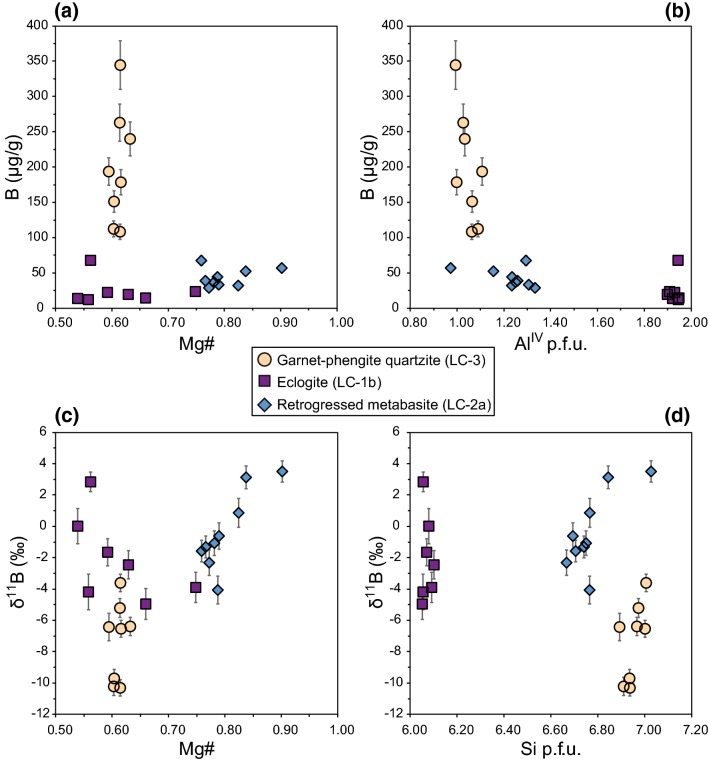
White mica boron geochemistry linked to major element chemistry. **a** [B] vs Mg#, **b** [B] vs Al^IV^ p.f.u., **c** δ^11^B vs Mg# and **d** δ^11^B vs Si p.f.u

Peak metamorphic phengite in the garnet–phengite quartzite has a relatively small range of values for Mg# and Si p.f.u. but highly variable B contents (Fig. 5a, b), suggesting that the boron concentrations in the individual phengite grains are governed by processes that are not reflected in the major element chemistry. Similarly, the δ^11^B values vary over 7% without accompanying variation in major elements (Fig. 5c, d), pointing to decoupling of boron isotope variations from major element mineral chemistry. Major element distribution maps also show that adjacent phengite grains are homogenous in their major element chemistry (supplementary Fig. 2). These observations are inconsistent with an inhomogeneous protolith, at least on the thin-section scale.

##### Retrograde overprint

The tight cluster of Si p.f.u. values at 6.95 ± 0.06 and the internal major element homogeneity of individual phengite grains (supplementary Fig. 1) do not support a significant retrograde overprint. The Si content of potassic white mica (muscovite/phengite) is positively correlated with pressure and indicates whether the mineral equilibrated at peak metamorphic conditions or partially recrystallized during a retrograde overprint (Warren et al. 2012), although absolute pressure conditions may only be derived when a limiting assemblage of K-feldspar + phlogopite + quartz (Massonne and Schreyer 1987) or garnet + kyanite + qz/cs (Krogh Ravna and Terry 2004) is present. In mineralogically similar garnet–phengite schists from the LCU, phengite inclusions in garnet also have high Si values (6.7–7.0 p.f.u) reflecting equilibration at UHP conditions, whereas matrix phengites record a much larger chemical variability (6.4–7.0 Si p.f.u.), which has been interpreted to reflect retrograde chemical reactions (Gouzu et al. 2006). The lack of variation in the major element chemistry, the similar textural features of all phengites analysed and the lack of retrograde mineral phases in the garnet–phengite quartzite point to equilibration under identical peak P–T conditions and the lack of any significant influence of a retrograde overprint.

##### Prograde growth zoning

**Fig. 6:**
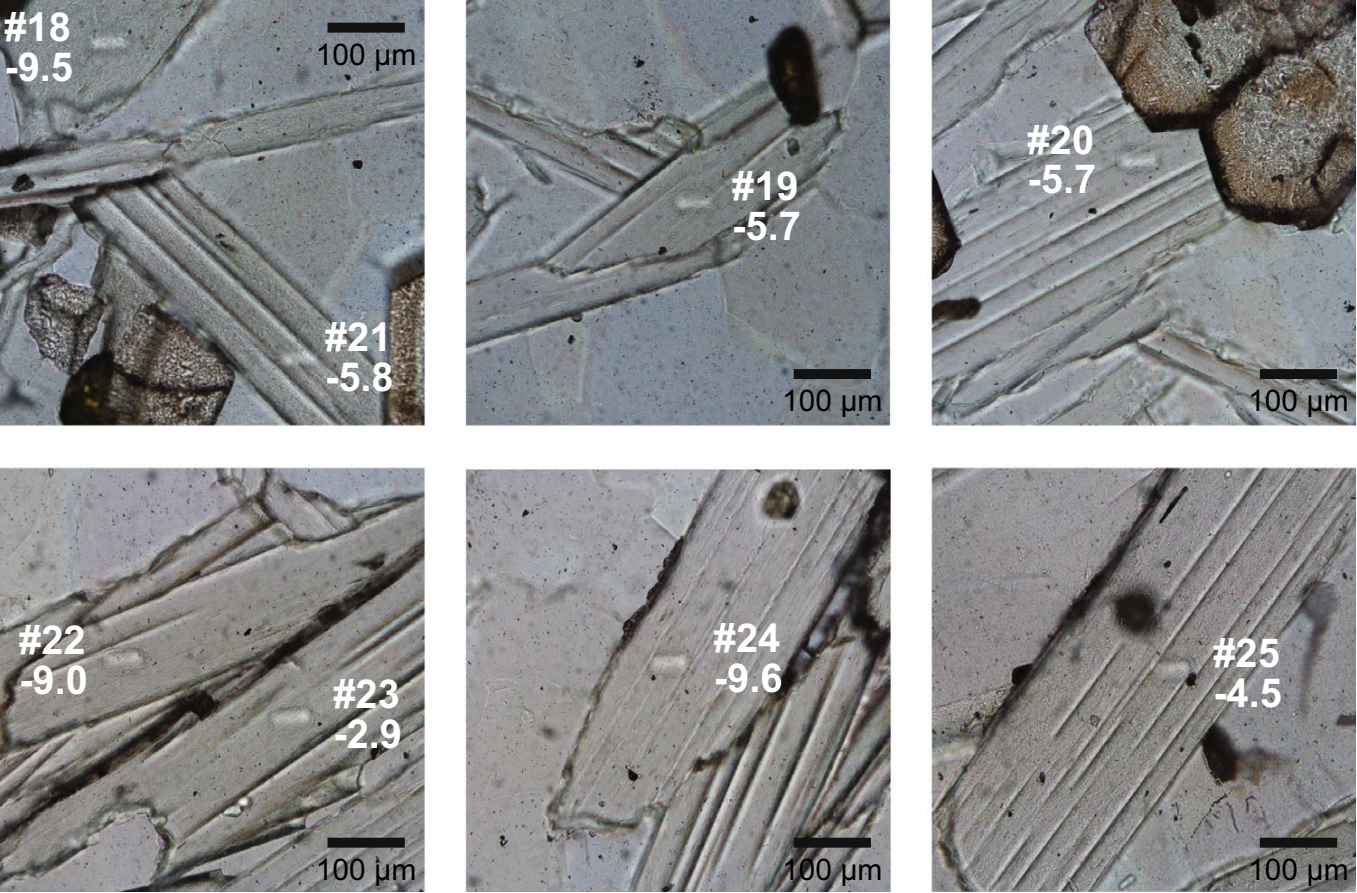
Ion microprobe spot locations in phengites from the garnet–phengite quartzite. Analysis number (see Table 2) and δ^11^B values are given next to the spot

Even though the major element composition of the various phengite grains in the garnet–phengite quartzite is relatively homogeneous (supplementary Figs. 1, 2), trace elements may be less homogeneous and can reflect distinct stages of growth zoning. Compositional zoning in trace elements has been shown to reveal complexities in the formation of minerals that were previously undetectable with petrography or major element data, since some trace elements are generally less vulnerable to diffusive resetting and can track the mineral reaction history that excludes the major elements (Konrad-Schmolke et al. 2008; Kohn 2014; Raimondo et al. 2017). White mica is able to preserve chemical signatures of their growth history and may even maintain ^40^Ar/^39^Ar ages reflecting discrete prograde stages of mineral growth (Bröcker et al. 1993; Putlitz et al. 2005). Chemical re-equilibration is dominantly controlled by fluid availability and intensity of deformation, and muscovite can partially re-equilibrate without affecting the microstructures (Airaghi et al. 2017). Studies on the behaviour of B in single metamorphic white mica crystals are rare and largely focused on distinct zones that show changes in both major and trace element chemistry related to fluid-induced overprint causing a decrease in B contents (Konrad-Schmolke et al. 2011; Halama et al. 2014). No distinct zonation is observed optically, in back-scattered electron images or in element distribution maps in phengites from sample LC-3. Moreover, the analysis spots in the different phengite crystals are all in a central position in grains with similar texture and major element chemistry (Fig. 6). Therefore, there is no indication of growth zoning in phengite.

##### Diffusion

For boron, only very few studies have evaluated high-temperature diffusion-induced B isotope fractionation (Kowalski and Wunder 2018). Experimental data show that kinetic B isotope fractionation is insignificant at melt crystallization temperatures (1200–1600 °C; Chakraborty et al. 1993). In natural samples, B abundances in mantle xenolith minerals do not vary significantly, consistent with expected low B diffusivities (Kaliwoda et al. 2008), and uniform compositions in deformed and undeformed tourmaline also indicate the absence of significant B diffusion (Büttner and Kasemann 2007). Hence, in accordance with Kowalski and Wunder (2018), B isotope fractionation from diffusion is not considered here.

##### Prograde devolatilization

Zoning in both [B] and δ^11^B has been studied extensively in metamorphic tourmaline. For instance, zoned tourmalines in metasedimentary rocks show decreasing δ^11^B values from core to rim (Nakano and Nakamura 2001; Bebout and Nakamura 2003; Berryman et al. 2017). This core-to-rim zoning pattern is thought to reflect progressive devolatilization of B during metamorphism (Bebout and Nakamura 2003). Boron originally present in white mica is mobilized into a fluid phase with ^11^B preferentially removed from the rock during prograde dehydration (Wunder et al. 2005). This results in a trend to lower δ^11^B values in mica and fluid, which is reflected in concurrently growing tourmaline (Bebout and Nakamura 2003; Berryman et al. 2017). The loss of B from white mica during prograde metamorphism and progressive devolatilization will lead to the formation of mica with more negative δ^11^B values.

**Fig. 7:**
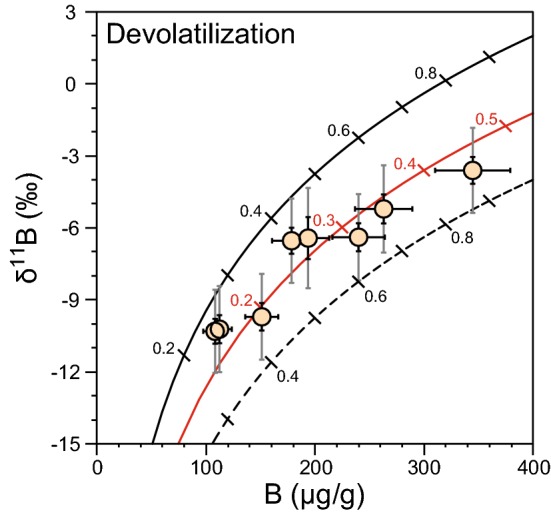
Rayleigh distillation model to simulate the compositional evolution during prograde devolatilization and associated loss of boron, accompanied by a decrease in δ^11^B values. The black curves start at initial values of [B] = 400 μg/g and δ^11^B =  + 2% (solid line) and − 4% (stippled line), respectively. The red curve starts at [B] = 750 μg/g and δ^11^B =  + 4%. Numbers denote the fraction of boron that remains in the rock after devolatilization. Error bars for δ^11^B values represent the external precision (black) and the accuracy (grey)

Here, we test whether the B systematics in the garnet–phengite quartzite reflect devolatilization of B during metamorphism. We test this model by a Rayleigh distillation calculation, using the formula:

$${\delta }^{11}{B}_{f}={\delta }^{11}{B}_{i}+1000\left({F}^{\left(\alpha -1\right)}-1\right),$$

where *δ*^*11*^*B*_*f*_ and *δ*^*11*^*B*_i_ are the final and initial B isotopic compositions of the rock, *F* is the fraction of B that remains in the rock after devolatilization, and *α* is the temperature-dependent fluid–mineral fractionation factor (Wunder et al. 2005). For the fluid–phengite fractionation factor, we use a value of 1.00833 reflecting a temperature of 600 °C. The other parameters that need to be constrained for the modelling are the initial B contents and δ^11^B values. We calculated two curves based on initial δ^11^B values of − 4% and + 2% and 400 μg/g B. However, higher initial values for both B and δ^11^B are also permitted by the data. For instance, a combination of *B* = 750 μg/g and δ^11^B =  + 4% would produce a devolatilization curve that fits the data points in a manner similar to the curves described above (Fig. 7).

The modelled fraction of B lost during devolatilization depends on the initial B content and δ^11^B value, both of which are unconstrained. The assumed initial B contents in phengite can be related to whole rock [B] using the relationship (B/K_2_O)_phe_ = (B/K_2_O)_WR_, where [K_2_O]_phe_ = 10 wt% and [K_2_O]_WR_ is assumed to be 1.55 wt% (Selverstone and Sharp 2013). Then, [B] content would vary between 60 and 116 μg/g, which overlaps with measured values for siliceous sedimentary rocks. We can also evaluate the unknown initial δ^11^B of the protolith by comparison with published data on sedimentary rocks. These cover a range of > 30%, from δ^11^B = − 24 to + 5% (Ishikawa and Nakamura 1993; Leeman et al. 2004; Romer et al. 2014; Tonarini et al. 2011). This considerable range of potential initial isotopic ratios in the protolith is problematic for assigning accurate initial parameters for modelling any kind of fluid–rock interaction (Romer et al. 2014). Restricting the protolith to known silica-rich rock compositions (siliceous ooze and chert) provides only a small limitation, as values vary from − 17.0 to + 8% (Ishikawa and Nakamura 1993; Kolodny and Chaussidon 2004). We note, however, that high positive values (δ^11^B > 4%) are rare and most values fall between − 8 and + 4%, suggesting that an initial starting point of δ^11^B =  + 4% serves as a reasonable upper limit. Model curves based on more extreme initial mica compositions (e.g. *B* = 1400 μg/g and δ^11^B =  + 10%) that also fit the data are, therefore, excluded.

The modelling of the phengite composition in the garnet–phengite quartzite indicates a loss of 15–75% B for an initial composition of 400 μg/g B and δ^11^B = 0 ± 2%, and a loss of 55–85% B if initial values of 750 μg/g B and δ^11^B =  + 4% are assumed (Fig. 7). For both modelled scenarios, the wide range of B contents and δ^11^B values in phengite is noteworthy, as this pattern in different whole rock samples would suggest different degrees of devolatilization due to different temperatures reached. In a single, well-equilibrated sample that contains chemically (Fig. 3) and texturally (Fig. 6) indistinguishable phengites, this explanation is untenable. The analysed spots are in mica grains of similar size with random orientations, and measurements were typically taken in central parts of the grain. There is no obvious relationship of [B] or δ^11^B with spot position or grain orientation (Fig. 6). Preservation of initial differences in B content of the individual phengite grains cannot be entirely excluded, but it does not seem to be a major factor based on the only small variations in all other mineral chemical parameters and the intra-grain and inter-grain major element homogeneity (supplementary Figs. 1, 2). Despite the good model fit to the data, the implicit large variations in devolatilization efficiency are unrealistically high and do not support the devolatilization model.

##### Peak metamorphic fluid–rock interaction

Since none of the processes discussed above satisfactorily explains the petrographic observations in combination with the variable [B]-δ^11^B data, we will now evaluate whether fluid–rock interaction with an intergranular fluid phase at or near peak metamorphic conditions can provide an answer. If such a model can be conceptually applied to the garnet–phengite quartzite, it implies that the fluid did not interact with all individual phengites to the same degree. Instead, some phengites retained their initial or near-initial B geochemical signatures, whereas other phengite grains were able to exchange B with the fluid for an extended period, leading to significant modifications in both [B] and δ^11^B. Variations in both [B] and δ^11^B have been extensively studied in metamorphic tourmaline, where the involvement of distinct fluids with distinct B isotope composition was used to explain tourmaline rims that are either lighter (Trumbull et al. 2009) or heavier (Marschall et al. 2008) than the respective cores during open system tourmaline crystallization.

For the fluid–rock interaction modelling, we use equations for open system fluid–rock interaction presented in Nabelek (1987). For B concentrations, the fluid/rock ratio *N* is determined as:

$$N=\left(\frac{1}{D}\right)\times \mathrm{l}\mathrm{n}\left[\frac{{C}_{f}^{i}-{C}_{r}^{i}D}{{C}_{w}^{i}-{C}_{r}^{f}D}\right],$$

where ${C}_{r}^{f}$ is the final concentration of the trace element in the rock, ${C}_{r}^{i}$ is the initial trace element concentration in the rock, ${C}_{w}^{i}$ is the initial trace element concentration in the fluid, *D* is the partition coefficient between fluid and rock. This equation is solved for ${C}_{r}^{f}$:

$${C}_{f}^{r}=\frac{{C}_{w}^{i}}{D}-\left(\frac{{C}_{w}^{i}}{D}-{C}_{r}^{i}\right)\times {e}^{-ND}.$$

For the boron partition coefficient between fluid and white mica, we use a value of 1.4 based on the work by Marschall et al. (2006a). The parameters ${C}_{r}^{i}$ and ${C}_{w}^{i}$ are adjusted to obtain a reasonable fit for the model curves. Stable B isotope exchange is modelled using an equation from Taylor (1977):

$$N=ln\left[\frac{{\delta }_{w}^{i}-{\delta }_{r}^{i}+\Delta }{{\delta }_{w}^{i}-{\delta }_{r}^{f}+\Delta }\right],$$

where *N* is the fluid/rock ratio, ${\delta }_{w}^{i}$ is the initial stable isotope ratio in the fluid, ${\delta }_{r}^{i}$ is the initial stable isotope ratio in the rock, ${\delta }_{r}^{f}$ is the final stable isotope ratio in the rock, and Δ is the equilibrium stable isotope fractionation between rocks and fluid (*Δ* = *δ*_*r*_ − *δ*_*w*_). This equation is solved for ${\delta }_{r}^{f}$:

$${\delta }_{r}^{f}=\left({\delta }_{w}^{i}+\Delta \right)-{e}^{-N}\left({\delta }_{w}^{i}-{\delta }_{r}^{i}+\Delta \right).$$

We assume a temperature of 600 °C for the peak metamorphic fluid–rock interaction, which results in a Δ^11^B of − 8.4% for neutral and acidic fluids (Wunder et al. 2005). The parameters ${\delta }_{w}^{i}$ and ${\delta }_{r}^{i}$ were varied, and eventually the open system exchange equations for the trace element B and the B isotope composition were combined.

**Fig. 8:**
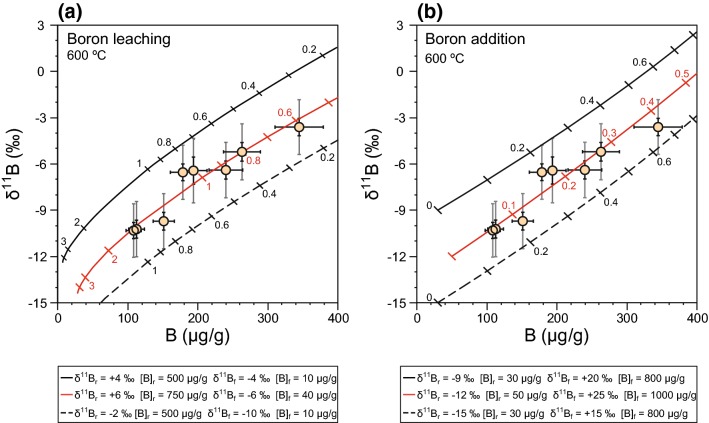
Fluid–rock interaction modelling for peak metamorphic overprint in the garnet–phengite quartzite (sample LC-3; beige circles). Two distinct scenarios are modelled: **a** boron leaching and **b** boron addition. Starting compositions of mica and fluid are given below the figures. Numbers at the curves mark the fluid/rock ratios. Error bars for δ^11^B values represent the external precision (black) and the accuracy (grey)

Two distinct scenarios of fluid–rock interaction are considered, assuming a temperature of 600 °C that is appropriate for peak metamorphic conditions (Fig. 8). In the first case, white mica is assumed to lose B, leading to successively lower δ^11^B values with increasing *N* (Fig. 8a). In the second case, white mica is assumed to gain B from a B-rich fluid (Fig. 8b). Fluid–rock interaction modelling provides suitable solutions for both scenarios, suggesting that it is an appropriate process to explain the [B]-δ^11^B variability in white mica. Since both modelled scenarios of fluid–rock interaction fit the data using appropriate parameters, the models themselves cannot distinguish which scenario is more likely. The models show that low fluid/rock ratios (≤ 1) are sufficient to cause significant variation in B concentration and B isotopic composition. Importantly, a scenario of small amount of intergranular fluid interaction with the rocks at (ultra)high pressures is consistent with the petrographic and textural evidence.

The range of parameters chosen to obtain a good model fit provide an indication about the likely fluid composition. For the first case (boron leaching; Fig. 8a), the initial fluid composition is approximately constrained to δ^11^B_fluid_ = − 7 ± 3% and [B] = 10–40 μg/g, using initial values for white mica of [B] = 500–750 μg/g and δ^11^B_mica_ =  + 2 ± 4% (Fig. 8a). These highly negative δ^11^B_fluid_ values imply residual rock compositions with even more negative δ^11^B values around − 12 to − 18% (at 600 °C) from which these fluids were derived. Such low δ^11^B values are at the lower end of δ^11^B values that have been observed in high-pressure metamorphic rocks, which typically range from − 15 to + 5% (Peacock and Hervig 1999; Nakano and Nakamura 2001; Pabst et al. 2012; Angiboust et al. 2014; Halama et al. 2014). If these values were reached in the subducting slab, near-complete preceding devolatilization would be required, leaving little fluid around to potentially interact with surrounding rocks.

In the second case (boron addition; Fig. 8b), a B-rich fluid with high positive δ^11^B values (δ^11^B =  + 20 ± 5) can constrain fluid–rock interaction models that encompass the data points. All of these boron addition models require only small fluid/rock ratios (≤ 0.6). High positive δ^11^B values are typical for slab serpentinites that formed by subduction fluid infiltration, covering a wide range in δ^11^B from + 7 to + 24 (Scambelluri and Tonarini 2012). Isotopic fractionation between serpentine and fluid is not well constrained, but minimal fractionation is expected if the pH value of the fluid is high and only at lower pH values would the fluid become isotopically lighter than the serpentinite (Benton et al. 2001), corresponding to a more positive δ^11^B in the fluid source rocks. However, serpentine dehydrates over a small temperature interval so that all fluid released is likely to be pooled and would then have the same B isotopic composition as the serpentine. Hence, the range of modelled δ^11^B_fluid_ values is in good agreement with serpentinite as fluid source rock. The high B contents are more problematic to explain, but high B contents (250 mg/l) were reported from fluids interpreted to reflect slab dehydration (Boschetti et al. 2017) and determined in melt inclusions (up to ca. 200 μg/g) thought to reflect a slab fluid influence (Jones et al. 2014). Serpentine minerals often have high B concentrations, typically around 10–100 μg/g (Benton et al. 2001; Vils et al. 2008), which makes them a suitable source for high-B fluids. Thermodynamic-geochemical modelling also suggest that breakdown of serpentine during subduction can release B-rich, high-δ^11^B fluids (Konrad-Schmolke and Halama 2014; Konrad-Schmolke et al. 2016). Moreover, the scenario of serpentinite-derived fluids fits with the Cignana peak P–T metamorphic conditions, which are similar to the expected antigorite breakdown (ca. 600–650 °C; Padrón-Navarta et al. 2013) and the abundant presence of serpentinites in the area. Hence, fluid–rock interaction at peak metamorphic conditions successfully explains the [B]–δ^11^B relationships, and serpentinite-derived fluid represents a likely candidate to account for this process. This interpretation implies that the fluid did not interact with all phengites to the same degree, but cause variable exchange of B with the fluid for different periods of time, leading to the observed variations.

#### Fluid–rock interaction during retrograde metamorphism

In both the eclogite and the retrogressed metabasite, white mica formed during post-peak metamorphic conditions. The breakdown of lawsonite, which is observed in the eclogite, is commonly caused by pressure decrease and/or temperature increase (Heinrich and Althaus 1988). For eclogites from Lago di Cignana, Groppo et al. (2009) concluded based on detailed petrographic observations and pseudosection modelling that lawsonite was part of the peak metamorphic assemblage and breakdown of lawsonite to epidote + paragonite occurred during decompression, reflecting a retrograde post-peak metamorphic assemblage. The paragonite in the eclogite has constant Si but variable Mg# (Fig. 5), suggesting equilibration with somewhat different proportions of neighbouring minerals during replacement of lawsonite and hence different effective bulk compositions of the equilibrated rock volume. However, intra-grain variability in major element composition is small and no clear zonation is discernible in element distribution maps (supplementary Fig. 3). Ion microprobe analysis spots were placed in central parts of texturally similar mica grains, so that any obvious influence of texture/position on the B geochemistry was avoided.

Phengite in the retrogressed metabasite is variable in both Mg# and Si p.f.u., indicating variable and extended recrystallization on the retrograde P–T path. The weak patchy zonation in Si, Al and Mg suggests only partial re-equilibration during retrogression leading to the preservation of chemically distinct mica domains (supplementary Fib. 4). A more variable chemical composition in secondary white mica that formed due to retrograde reactions compared to homogeneous primary, peak metamorphic white mica has been also observed in a subduction-related HP metagabbro that preserves an eclogite-facies assemblage (Putlitz et al. 2005). δ^11^B in phengite shows a weak positive correlation with Mg# and Si, but a clear correlation of [B] with major element chemical parameters is absent.

Both rocks had a mafic igneous rock as protolith. They also reached similar peak P–T conditions, but given the effects of retrograde metamorphism it is unlikely that effects of prograde and peak metamorphic processes are recorded in the [B]-δ^11^B systematics. For instance, the lack of a systematic correlation between [B] and δ^11^B (Fig. 4) and the large spread in δ^11^B values (ca. 9%) over a small interval in [B] are difficult to reconcile with devolatilization trends or prograde zonation. Instead, we will test and model the effects of fluid–rock interaction on the B systematic of these rocks, using the equations presented earlier in the text.

**Fig. 9:**
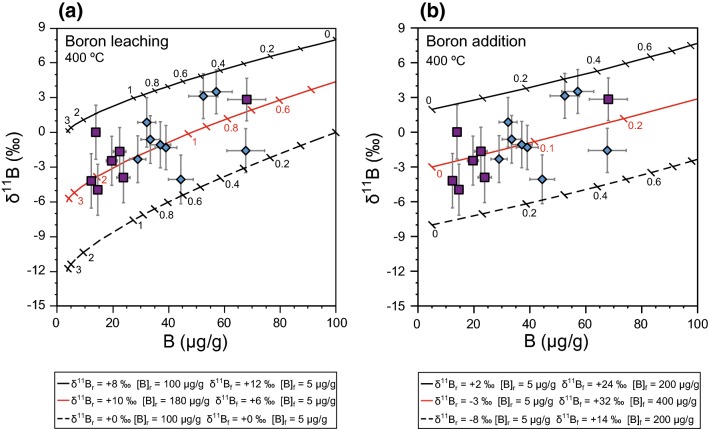
Fluid–rock interaction modelling for retrograde metamorphic overprint in samples LC-1b (purple squares) and LC-2a (blue diamonds). Two distinct scenarios are modelled: **a** boron leaching and **b** boron addition. Starting compositions of mica and fluid are given below the figures. Numbers at the curves mark the fluid/rock ratios. Error bars for δ^11^B values represent the accuracy (grey)

We assume a temperature of 400 °C for the retrograde overprint, which results in a Δ^11^B of − 12.0% for neutral and acidic fluids (Wunder et al. 2005). The parameters ${\updelta }_{\mathrm{w}}^{\mathrm{i}}$ and ${\updelta }_{\mathrm{r}}^{\mathrm{i}}$ were varied, and eventually the open system exchange equations for the trace element B and the B isotope composition were combined. The B partition coefficients for fluid/phengite (1.4) and fluid/paragonite (0.9) are slightly different (Marschall et al. 2006a). They are derived from a combination of clinopyroxene/fluid partition coefficients (Brenan et al. 1998) and inter-mineral partition coefficients (Marschall et al. 2006a). The relative uncertainties in these inter-mineral partition coefficients are ~ 39% for clinopyroxene/phengite (0.0026 ± 0.010, *n* = 5) and ~ 22% for clinopyroxene/paragonite (0.015 ± 0.003, *n* = 2), respectively. Assuming an overall relative uncertainty of 40% for fluid/mica partition coefficients, values for fluid/phengite and fluid/paragonite overlap and for clarity all models were calculated using the fluid/phengite partition coefficient. The model curves shown (Fig. 9) encompass all data points. As for the garnet–phengite quartzite, two different sets of models were developed.

The initial fluid composition in the first set of models, reflecting boron leaching (Fig. 9a), can be approximated as δ^11^B_fluid_ =  + 6 ± 6% and [B] = 5 μg/g. With these parameters, the data can be successfully modelled for values of *N* ranging from ~ 0.2 to ~ 3. The second set of models, reflecting addition of boron (Fig. 9b), constrains the initial fluid to very high δ^11^B values (+ 14 to + 32) with high B contents of 200–400 μg/g (Fig. 9b). All data points can be explained with low fluid/rock ratios (*N* < 0.5).

The [B]-δ^11^B systematics in white mica from the Catalina Schist were linked to fluids from highly devolatilized (low δ^11^B) and less devolatilized (moderate δ^11^B of − 2 to + 8%) rocks (Sievers et al. 2017). Depletion of B during fluid–rock interaction has also been observed in the HP rocks from the Sesia zone where a percolating fluid caused a decrease in [B] in overprinted phengite rims (Konrad-Schmolke et al. 2011; Halama et al. 2014). The range in δ^11^B for the shallow fluids from moderately devolatilized rocks overlaps well with the initial δ^11^B_fluid_ in the first set of model calculations. King et al. (2007) proposed a range in δ^11^B between + 2% at 500 °C and + 9% at 300 °C for slab-derived fluids, overlapping the B isotopic compositions of the fluids in our models (Fig. 9a). Tourmalines from Lago di Cignana metasedimentary rocks show an increase of δ^11^B values to + 4%, which resulted from retrograde influx of B by fluids (Bebout and Nakamura 2003). Since the B isotope fractionation between tourmaline and fluid is relatively small (Δ^11^B_tourmaline-fluid_ = − 2.7% at 400 °C; Meyer et al. 2008), these tourmaline δ^11^B values correspond to δ^11^B_fluid_ values of around + 7, in perfect agreement with our modelled fluid compositions (Fig. 9a). In contrast, the second set of calculations (Fig. 9b) requires at residual rocks with highly positive δ^11^B, which would be in line with a serpentinite-derived fluid but not with fluids derived from typical metabasites or metapelites. Serpentinites, however, are dehydrating at temperatures above 600 °C on the prograde metamorphic path, so it is difficult to envisage how serpentinite would be able to release fluid on the retrograde path. Therefore, the boron addition model does not seem feasible for the retrograde white mica, and instead boron leaching from the rocks is our preferred interpretation.

The key observation derived from the modelling is that small to moderate fluid/rock ratios are sufficient to cause significant shifts in [B] and the moderate decrease in δ^11^B_mica_. This remains true even when the significant uncertainties in some of the variables are taken into account. Rather than putting too much weight on the uncertainties in the model parameters it is worth emphasizing that the general process of fluid–rock interaction is suitable to explain the B geochemistry systematics of white mica in retrograde overprinted (U)HP metamorphic rocks and consistent with the petrographic and petrologic constraints. We, therefore, suggest that retrograde white mica and tourmaline rims record a similar overprint by the same kind of fluids.

## Conclusions

The white mica B concentration and isotopic data of the three Lago di Cignana (U)HP samples that have experienced a similar P/T evolution in a subduction zone setting reveal significant variability both between and within the samples. Phengite in a metasedimentary garnet–phengite quartzite has the highest B contents, reflecting B enrichment coupled too low to moderate K_2_O contents in the siliceous protolith. The decrease in δ^11^B with decreasing [B] cannot be explained by textural differences between grains, prograde growth zoning, diffusion or retrogression. The trend can be successfully modelled using a Rayleigh distillation equation, simulating devolatilization of B during prograde metamorphism, but this process fails to explain the wide range in [B] and δ^11^B in well-equilibrated rocks with a population of petrographically and texturally indistinguishable phengite. Alternatively, we modelled fluid–rock interaction at peak metamorphic conditions and several models successfully mirror the arrangement of data points. Our preferred model invokes fluid–rock interaction with a serpentinite-derived, high-B, high-δ^11^B (δ^11^B =  + 20 ± 5) fluid at or near peak metamorphic conditions. The [B]-δ^11^B variations in two samples that show a clear retrograde metamorphic overprint can be modelled by fluid–rock interaction at low to moderate (< 3) fluid/rock ratios during retrogression. The modelled fluid composition yields a moderately positive δ^11^B of + 6 ± 6, which agrees well with slab-derived fluids generally and those for Lago di Cignana in particular. Our observations underline the value of in situ observations of fluid–rock interaction processes in HP metamorphic rocks, which in turn is crucial for our understanding of the links between metamorphic processes in subduction zones and the geochemistry of arc magmatic rocks.

## Electronic supplementary material

Below is the link to the electronic supplementary material.

Supplementary file1 (PDF 12266 kb)
