# Supplementary material for: Boron isotope record of peak metamorphic ultrahigh-pressure and retrograde fluid–rock interaction in white mica (Lago di Cignana, Western Alps)
Source: Contrib Mineral Petrol. 2020 Feb 6;175(3):20. doi: 10.1007/s00410-020-1661-8 (PMC7010363; doi:10.1007/s00410-020-1661-8)

Supplementary Fig. 1:
Back-scattered electron (BSE) image and element distribution maps for a phengite in the garnet-phengite quartzite (sample LC-3). Boron and major element chemical data are from Table 2.

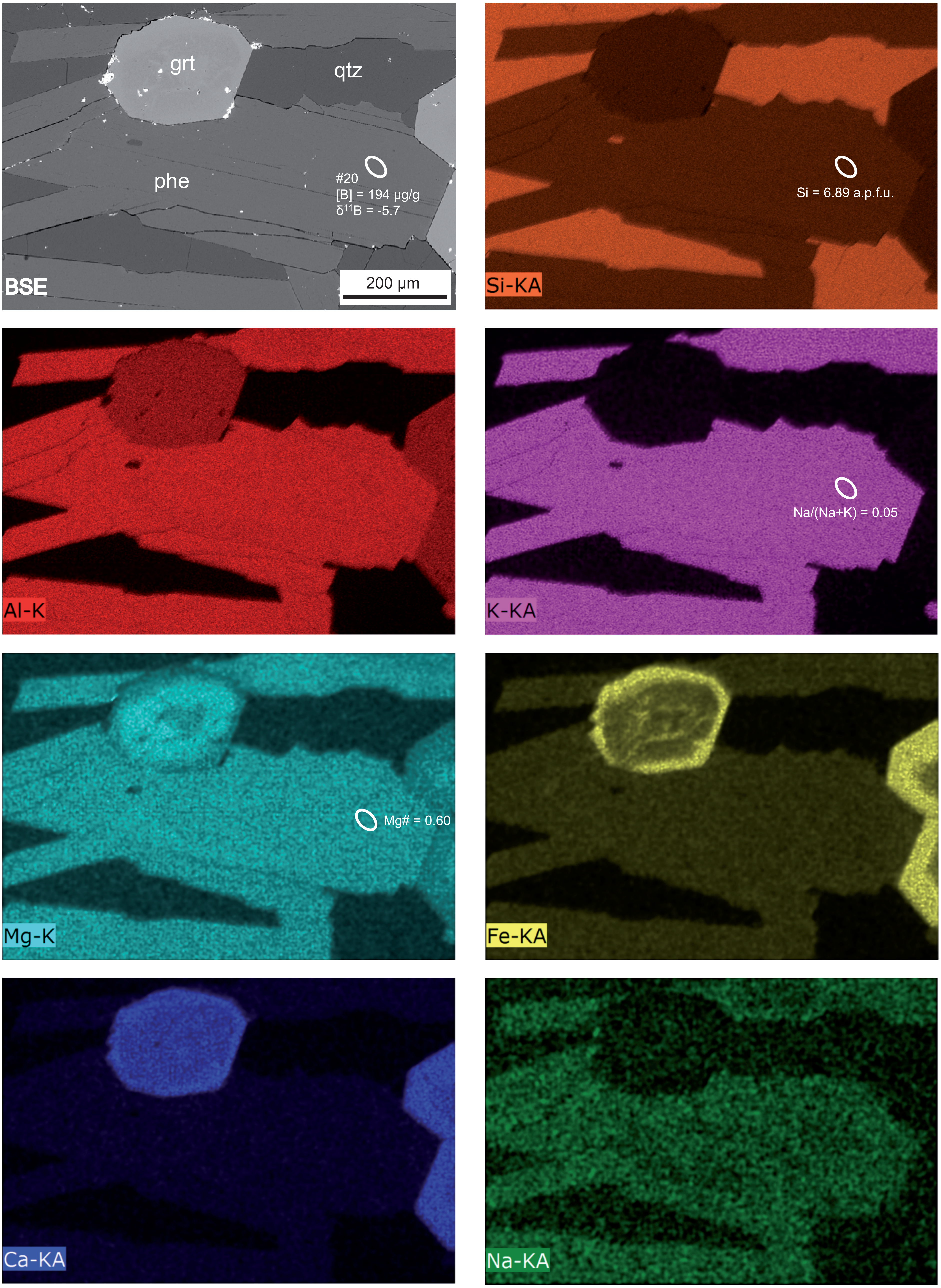

Supplementary Fig. 2:
BSE image and element distribution maps for phengites in the garnet-phengite quartzite (sample LC-3). Boron and major element chemical data are from Table 2.

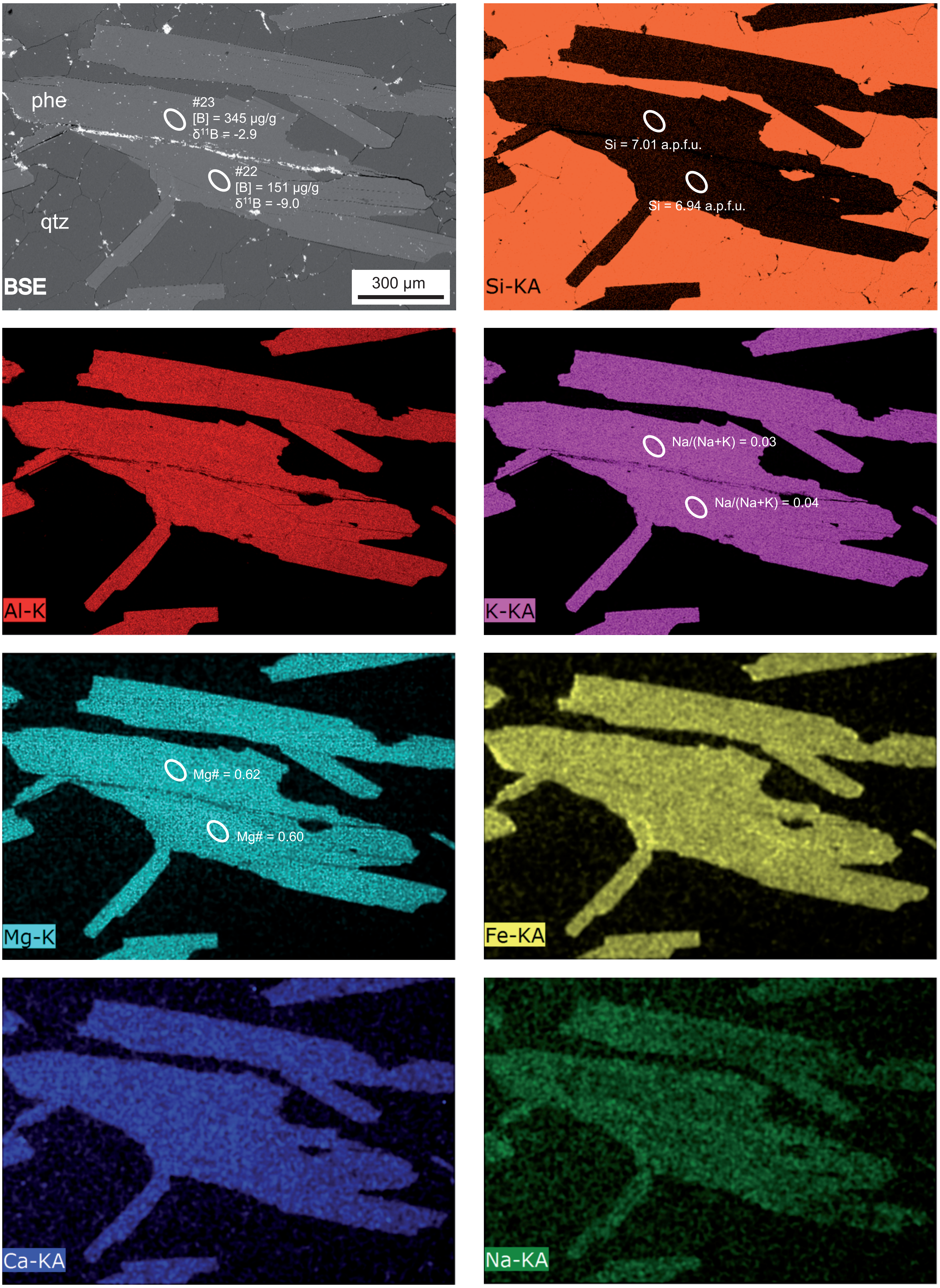

Supplementary Fig. 3:
BSE image and element distribution maps for a paragonite in the eclogite (sample LC-1b). Boron and major element chemical data are from Table 3.

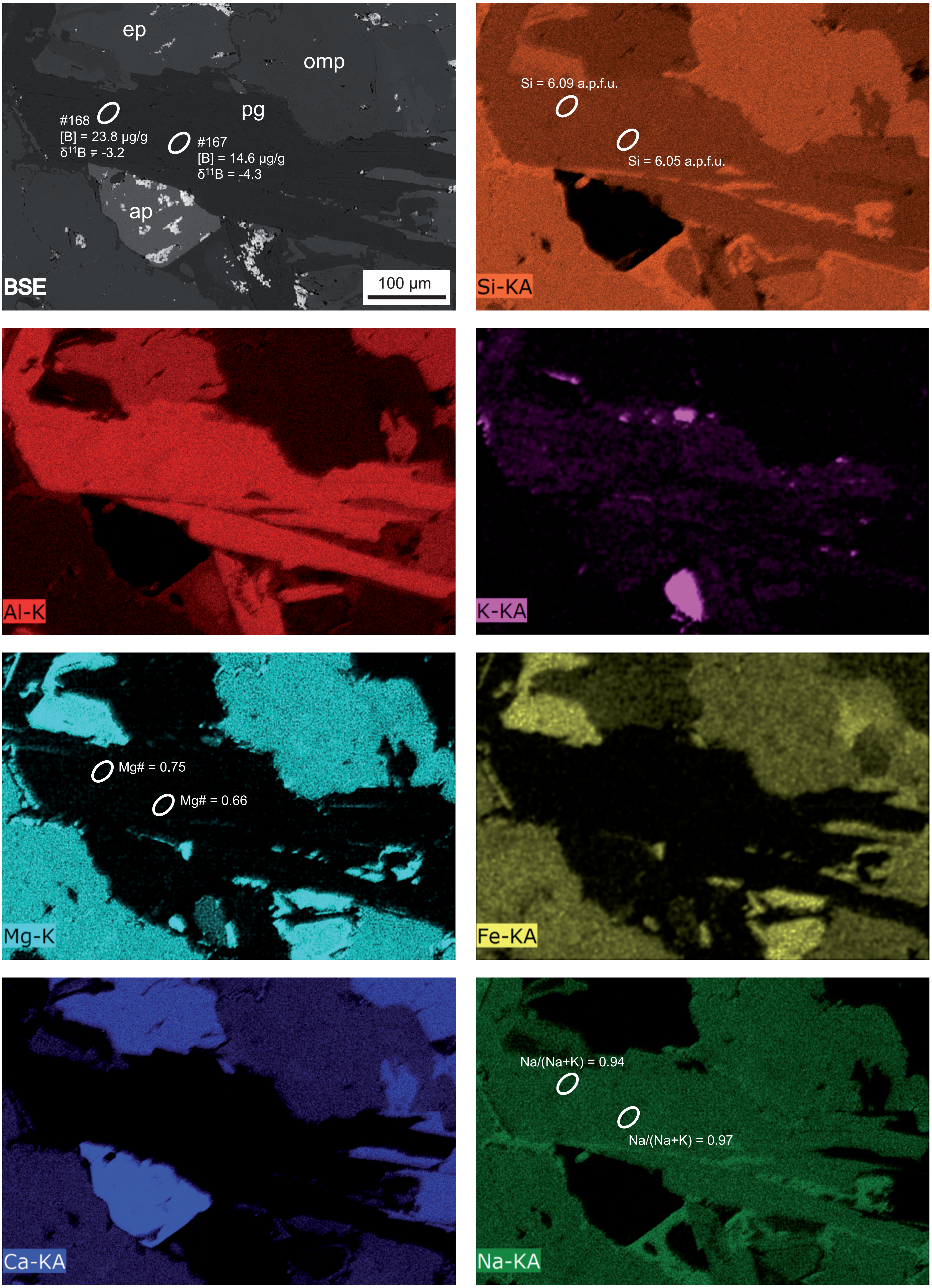

Supplementary Fig. 4:
BSE image and element distribution maps for phengites in the retrogressed metabasite (sample LC-2a). Boron and major element chemical data are from Table 4.

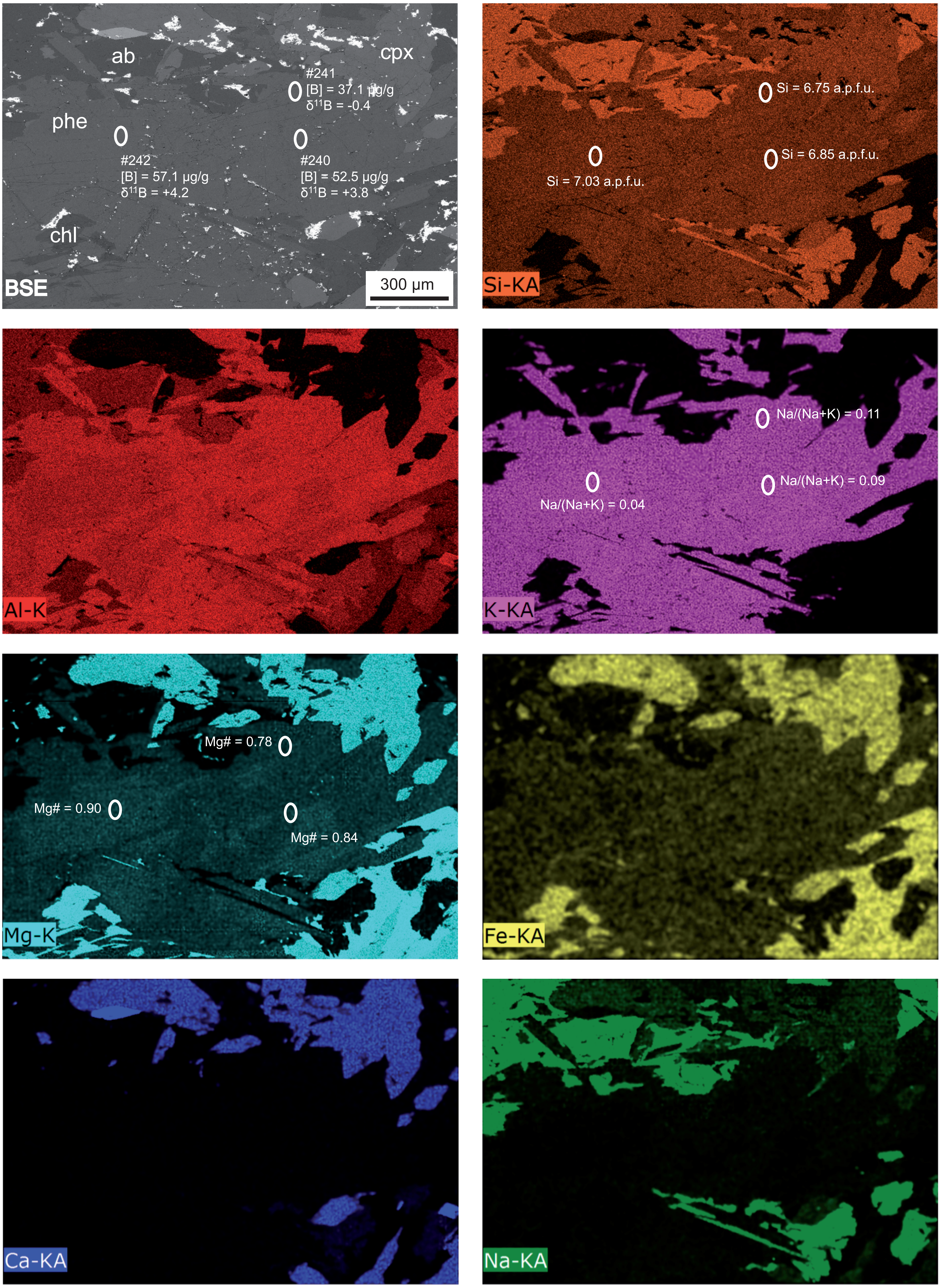

Supplement: Supplementary file 1 — Supplementary file1 (PDF 12266 kb) [file 410_2020_1661_MOESM1_ESM.pdf]
